# Development of an inducible mouse model of iRFP713 to track recombinase activity and tumour development *in vivo*

**DOI:** 10.1038/s41598-017-01741-0

**Published:** 2017-05-12

**Authors:** Andreas K. Hock, Eric C. Cheung, Timothy J. Humpton, Tiziana Monteverde, Viola Paulus-Hock, Pearl Lee, Ewan McGhee, Alessandro Scopelliti, Daniel J. Murphy, Douglas Strathdee, Karen Blyth, Karen H. Vousden

**Affiliations:** 1 Cancer Research UK Beatson Institute, Switchback Road, Glasgow, G61 1BD UK; 2 Institute of Cancer Sciences, University of Glasgow, Switchback Road, Glasgow, G61 1QH UK

## Abstract

While the use of bioluminescent proteins for molecular imaging is a powerful technology to further our understanding of complex processes, fluorescent labeling with visible light fluorescent proteins such as GFP and RFP suffers from poor tissue penetration and high background autofluorescence. To overcome these limitations, we generated an inducible knock-in mouse model of iRFP713. This model was used to assess Cre activity in a Rosa Cre–ER background and quantify Cre activity upon different tamoxifen treatments in several organs. We also show that iRFP can be readily detected in 3D organoid cultures, FACS analysis and *in vivo* tumour models. Taken together we demonstrate that iRFP713 is a progressive step in *in vivo* imaging and analysis that widens the optical imaging window to the near-infrared spectrum, thereby allowing deeper tissue penetration, quicker image acquisition without the need to inject substrates and a better signal to background ratio in genetically engineered mouse models (GEMMs).

## Introduction

**Figure 1:**
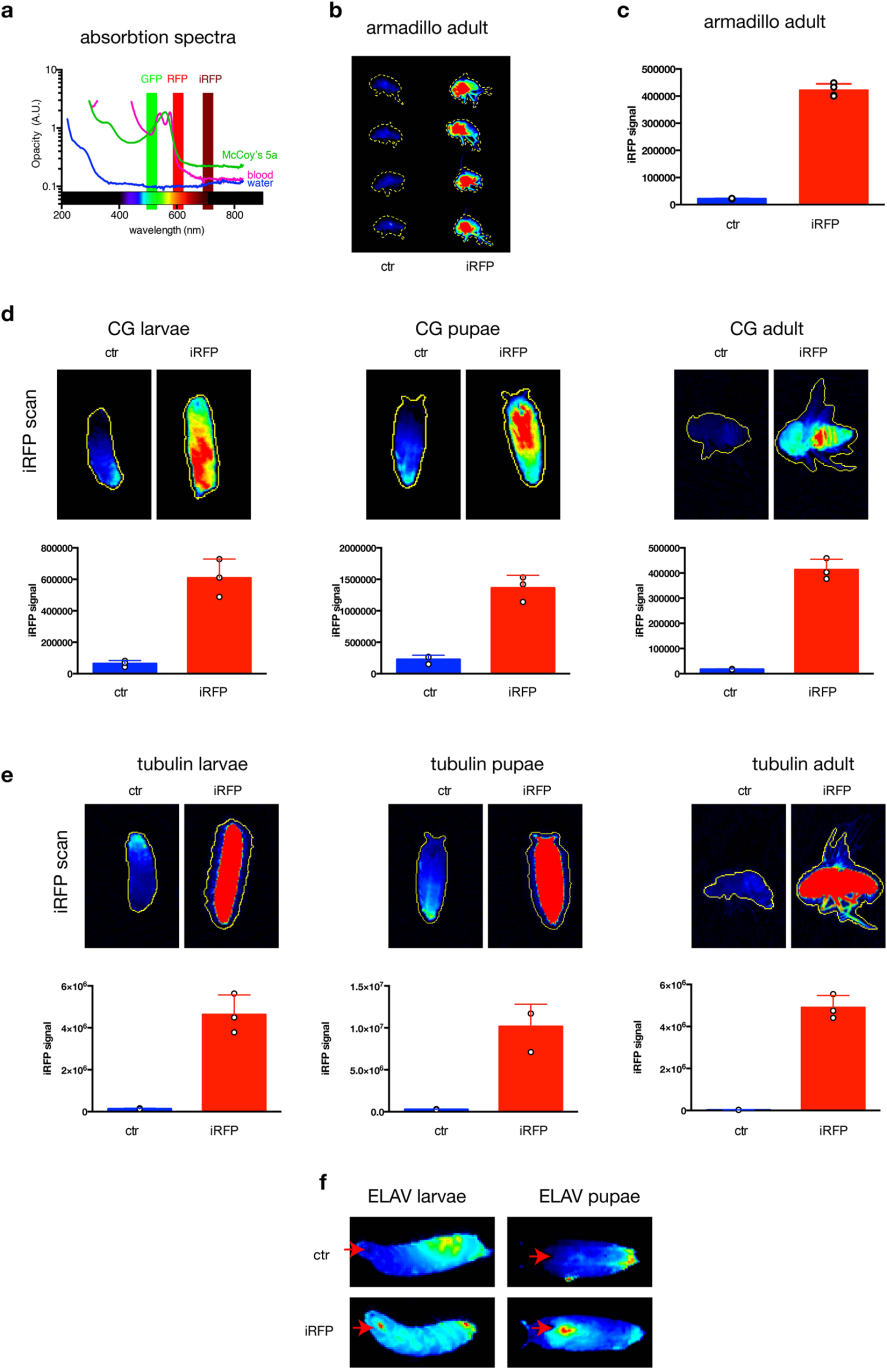
Generation of a drosophila iRFP *in vivo* model. (**a**) Absorption of cell culture media, water and blood. Excitation and emission wavelengths of commonly used FPs and iRFP have been highlighted. (**b**) iRFP introduced to a *Drosophila* armadillo driver strain was detected using a LI-COR Odyssey imaging system (**b**) and quantified in (**c**). (**d**) iRFP in larvae, pupae and adult CG driven *Drosophila* was detected using a LI-COR Odyssey imaging system and quantified in (**d**) lower panels. Each point represents an individual animal. Bar represents mean, error bar SD. 3 animals per condition were used. Identical look up tables (LUTs) were used for control and iRFP positive animals. (**e**) iRFP in larvae, pupae and adult tubulin driven *Drosophila* was detected using a LI-COR Odyssey imaging system and quantified in lower panels. Each value represents an individual animal. Bar represents mean, error bar SD. 3 animals per condition were used. Identical LUTs were used for control and iRFP positive animals. (**f**) iRFP in larvae, pupae and adult ELAV *Drosophila* was detected using a LI-COR Odyssey imaging system.﻿ T﻿he red arrow ﻿highlights﻿ the CNS. Identical LUTs were used for control and iRFP positive animals.

Imaging fluorescent and bioluminescent proteins is a keystone technique to quantify a wide variety of biological processes. Luciferase, green and red fluorescent proteins (GFP, RFP) are widely used as genetically encoded reporters to assess many aspects of cell behavior, including cell proliferation and tumour development both *in vitro* and *in vivo*. These methods allow for longitudinal studies without the need for invasive procedures or terminal sampling. Despite these clear advantages, use of probes that fluoresce in the visible light spectrum (such as RFP and GFP) have limited utility *in vivo* because they have poor tissue penetration and high autofluorencence. These limitations are reduced in proteins fluorescing in the near-infrared (NIR) spectrum, ranging from 700–900 nm (Fig. 1a). Several near-infrared fluorescent proteins have been developed. One of these, IFP1.1, was based on a bacteriophytochrome from *Deinococcus radiodurans* and although it is usable for *in vivo* applications, IFP 1.1 requires exogenous biliverdin to generate a clear image^1^.

Other versions of near infrared fluorescent proteins based on a different bacteriophytochrome (RpBphP2) do not depend on additional biliverdin, and these have been successfully introduced in several fields of research including 2D tissue culture, 3D assays and *in vivo* imaging of xenograft tumours^2–5^. To date, a whole family of iRFP proteins with distinct optical characteristics (reviewed in ref. 6) that allow spectral unmixing^4, 7^, two-color multimodal imaging^8^ and bimolecular fluorescence complementation (BiFC)^9^ have been developed. iRFP713 (subsequently referred to as iRFP) performs particularly well in these applications compared to visible light fluorescent proteins due to better signal to background ratio, the capacity for repetitive imaging after short time intervals without injecting a substrate (as required for luciferase) or a tracer (as required for PET scanning). Transgenic mice constitutively expressing iRFP﻿ were born at the expected mendelian ratios and fluorescence could be detected without exogenous biliverdin in several tissues^10^. However, targeted knock-ins of inducible near-infrared fluorescent proteins, which would allow monitoring of the fate of the cells in tissue of interest over time, have not yet been reported.

## Results

Although iRFP is a well-studied fluorescent protein, the effects of full body expression are so far only characterized in a constitutive mouse model^10^, which does not allow any control of where and when the iRFP is expressed *in vivo*. Prior to developing an inducible knock-in mouse model in the near-infrared spectrum, we confirmed that iRFP expression can be robustly detected in a *Drosophila* model without a requirement for exogenous biliverdin. As expected, we did not observe any reduction in the eclosion rate of *Drosophila* expressing iRFP under the control of the (ubiquitous) armadillo driver (Supplementary Fig. 1). iRFP was clearly detected in the adult (Fig. 1b, c) in an odyssey near-infrared imager. To test if iRFP is also detectable by microscopy, we imaged the eye and could clearly detect iRFP fluorescence compared to control animals (Supplementary Fig. 1, right panel). Furthermore, we were able to image and quantify iRFP in larvae, pupae and adult *Drosophila* using the CG (fat body) (Fig. 1d) and tubulin (ubiquitous high expression) (Fig. 1e) drivers. For both drivers, the iRFP signal is clearly detectable and consistent among different animals. To determine whether iRFP can also be detected using drivers targeted to small cell populations, we imaged ELAV^11^ (neuronal driver) iRFP and control *Drosophila* at different stages in development. As shown in Fig. 1f, even the relatively small mass of *Drosophila* central nervous system (CNS) can be detected in live specimens. Taken together this demonstrates that iRFP expression is well tolerated in *Drosophila* and clearly detectable at all stages of *Drosophila* development without exogenous biliverdin.

**Figure 2:**
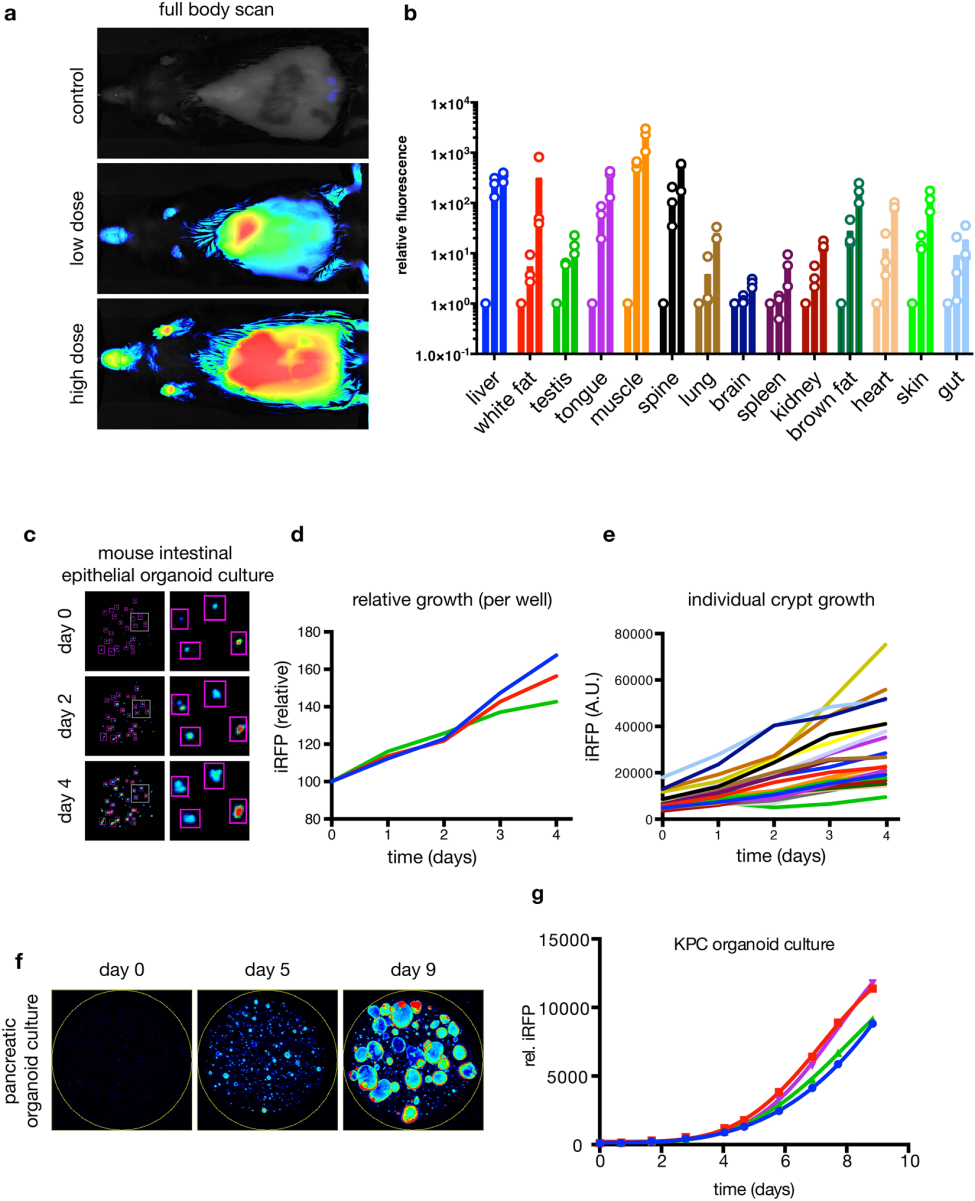
Generation of an inducible iRFP knock-in mouse. (**a**) Scans from LI-COR PEARL imaging system of control, low or high dose tamoxifen induced R26CreERT2; LSL-iRFP mice. (**b**) Quantification of individual resected organs on a LI-COR Odyssey. R26CreERT2; LSL-iRFP mice were un-induced (control) or treated with a low or high dose of tamoxifen, sacrificed and the organs resected. First bar: control; second bar: low dose; third bar: high dose. Mean relative to control values (**c**) of 3 mice were plotted (bar) each point represents one specimen (3 per condition). (**c**,**d**,**e**) Primary mouse intestinal organoid cultures of R26CreERT2; LSL-iRFP animals were prepared and induced with 100 nM 4OHT. Images of representative crypts (**c**, identical LUTs) and per well quantification (**d**) as well as individual crypt quantification (**e**) are shown. (**f**,**g**) Pdx1-Cre; LSL-iRFP; KPC pancreatic tumour organoids were cultured and quantified daily. Example images are shown in (**f**) (identical LUTs) and quantification of 4 replicate wells is shown in (**g**).

To establish an inducible system for iRFP expression in a mammalian model, we introduced *Irfp* under the control of a lox-stop-lox (LSL) element into the endogenous *Hprt* locus (Supplementary Fig. 2) of mouse embryonic stem cells (mESC). In this system the expression of iRFP is under the control of the endogenous *Hprt* promoter. Using these mESCs, we were able to verify that iRFP fluorescence was detectable upon adenoviral delivery of Cre recombinase (Supplementary Fig. 3) without supplementation of biliverdin to the medium. Encouraged by the signal obtained from a single copy of iRFP, we generated a Hprt^tm1(CAG-LSL-iRFP)^ (LSL-iRFP) mouse strain, crossed it to Gt(ROSA)26Sor^tm2(cre/ERT2)Brn^ (R26CreERT2) and induced the mice with either a single dose (Fig. 2a middle panel) or 4 doses of tamoxifen (Fig. 2a lower panel). As expected, the iRFP signal could be detected *in situ* and correlated well with tamoxifen induction (Fig. 2a and Supplementary Fig. 4). Consistent with previous observations^10^, no side effects or health concerns were detected in iRFP expressing animals after tamoxifen induction. Next, we measured iRFP intensity in isolated organs, to determine how efficiently R26CreERT2 recombinase is induced in different tissues by the tamoxifen regimes. While we could observe a dose dependent increase in relative iRFP signal (Fig. 2b), the level of induction over background varied from tissue to tissue. In the liver (where tamoxifen is metabolized into the active compound) a single dose induces maximum iRFP activity (Fig. 2b), indicating highly efficient Cre recombination in this organ. Most other tissues showed a robust induction of iRFP in response to Cre-induction, with the exception of the brain (Fig. 2b), where the iRFP increase was minimal. These results are consistent with previous work showing that while iRFP can be visualized in the brain if overexpressed^12^ R26CreERT2 expression is minimal in the adult mouse brain^13^ and would therefore not induce expression of iRFP.

Ectopically expressed iRFP has been shown to be a reliable marker to quantify cell growth in 2D and soft agar assays^2^. In order to determine if the protein expressed from a single *Irfp* allele is sufficient to detect and follow cell growth in primary organoids, we generated primary mouse intestinal epithelial organoid cultures (Fig. 2c) and quantified iRFP fluorescence per well (Fig. 2d) and per crypt (Fig. 2e). The signal increased over time in both cases and was clearly detectable at time of seeding. We were also able to measure growth by iRFP quantification in cultured pancreatic tumour organoids grown in matrigel (Fig. 2f and g), demonstrating that a single copy of iRFP is suitable to monitor growth of individual and pools of organoids without addition of exogenous biliverdin.

**Figure 3:**
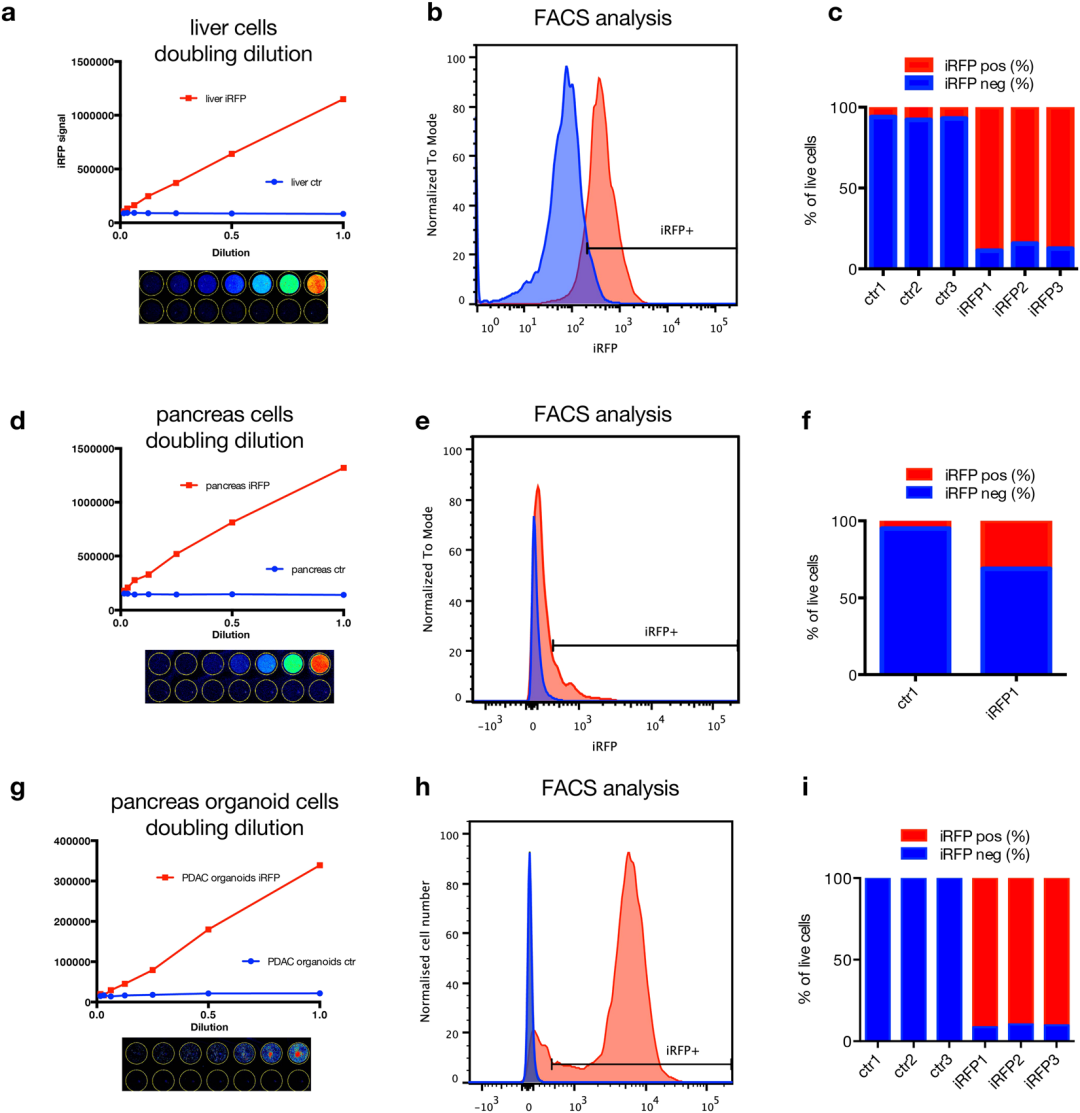
iRFP is detectable by FACS analysis. (**a**) iRFP positive or control livers were strained, prepared in a doubling dilution, scanned and quantified. Cells were subsequently FACS analyzed in (**b)**. (**c**) Quantification of iRFP positive cells from (**b)**. (**d**) P48-Cre; LSL-iRFP; KPC pancreatic tumour sample or an iRFP negative control pancreas were digested, strained and prepared in a doubling dilution. (**e**) These cells were FACS analysed and plotted by iRFP intensity. (**f**) Quantification of iRFP positive cells from (**e**). Pdx1-Cre; LSL-iRFP; KPC pancreatic tumour organoids were cultured and scanned (**g**). (**h**) Single cells derived from organoids were FACS analysed and plotted by iRFP intensity. (**i**) Quantification of iRFP positive cells from (**h**).

To investigate whether iRFP fluorescence is sufficiently bright to be detected directly in single cells from tissue, we isolated single cells from livers of iRFP expressing or control mice and analysed iRFP fluorescence in a doubling dilution series (Fig. 3a). Here we could show a linear relationship between the iRFP signal and the dilution factor, demonstrating that iRFP signal is a good marker for cell numbers over a wide range of concentrations. As expected, FACS analysis also showed a clear shift in iRFP expressing cells compared to control cells (Fig. 3b,c) demonstrating that iRFP can be readily detected in tissue-derived cells without the addition of biliverdin.

Next we prepared single cells from an iRFP positive pancreatic tumour and an iRFP negative control pancreas, and analyzed them in a doubling dilution (Fig. 3d) and by FACS (Fig. 3e,f). Interestingly, while we could clearly see the linear relationship between iRFP signal to cell number, only around 25% of the cells were iRFP positive, reflecting the heterogeneous nature of pancreatic tumours, where the majority of cells are derived from the stroma and therefore are not iRFP positive. When organoids of PDAC cells were analyzed the same way (Fig. 3g–i), they showed a strong and uniform shift to iRFP positivity.

**Figure 4:**
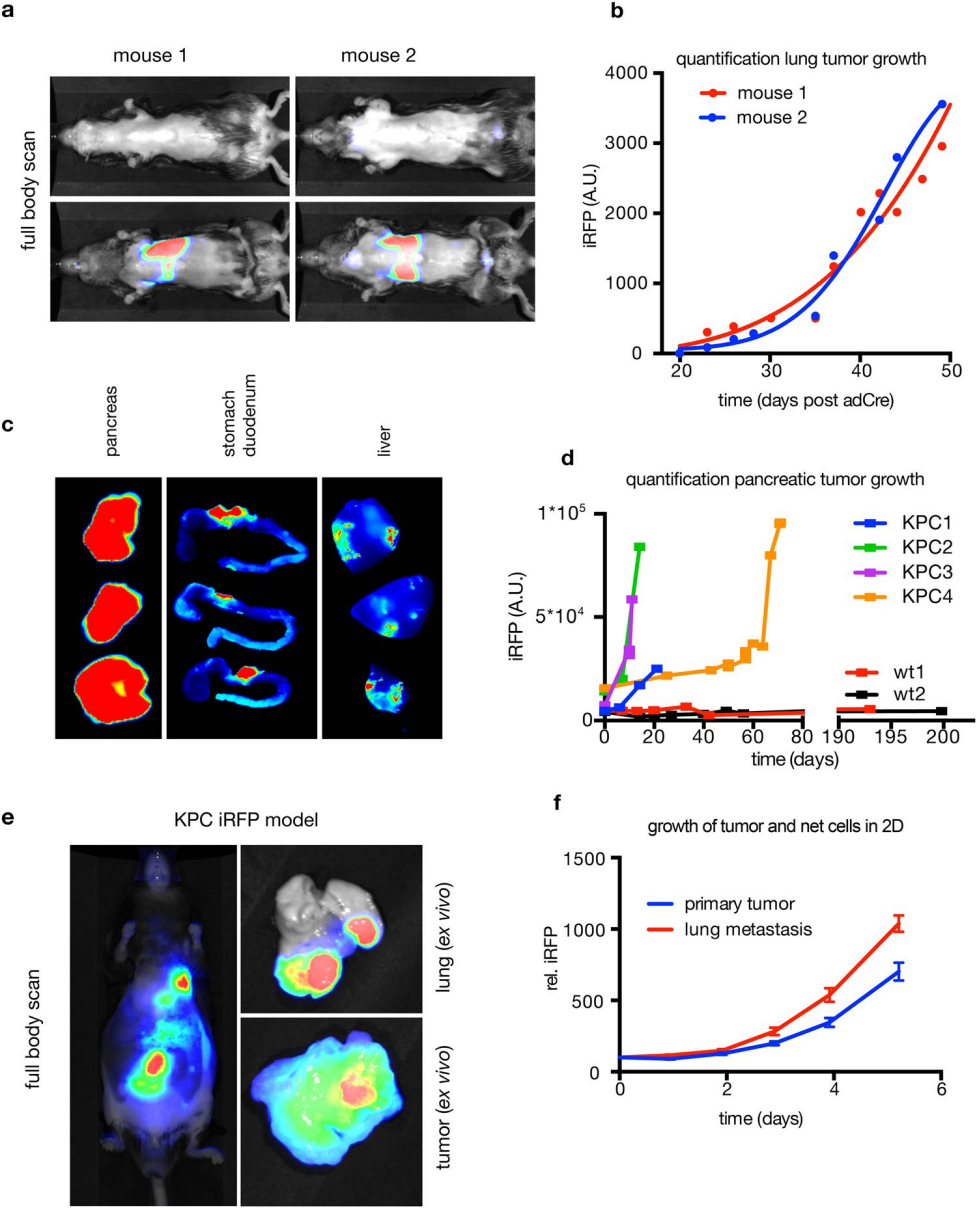
Analysis of tumour development in lung and pancreas by LSL-iRFP. (**a**) LSL- KRas^G12D^; Rosa26-LSL-MYC; LSL-iRFP mice were induced intranasally with Adeno-Cre and regularly imaged by PEARL. Images at day 20 post induction (upper) and endpoint (lower) are shown with identical LUTs. (**b**) The iRFP signal was quantified and plotted as baseline corrected value﻿s. (**c**) LI-COR Odyssey images of pancreas, gut and liver tissue of three Pdx1-Cre; LSL-iRFP mice. Identical LUTs were used for pancreas and stomach/duodenum. Liver tissue was detected at higher resolution. (**d**) Pdx1-Cre; LSL-iRFP (wt) and Pdx1-Cre; LSL-iRFP; KPC with Cre-inducible versions of 172H mutant p53 and oncogenic K-ras were imaged at the indicated time-points, 0 equals first day of imaging (average age approx. 8 weeks), and quantified until endpoint was reached for KPC animals. Wt animals were imaged again after approx. 200 days to exclude long-term changes in the iRFP signal. (**e**) Pdx1-Cre; LSL-iRFP; KPC mouse with detectable lung metastasis in the near-infrared image. Tumour and metastasis were resected and reimaged to confirm the source of the signal. The tissue was subsequently taken into culture, plated and quantified over time (**f**). Mean and SD (error bars) of 48 replicate wells are shown.

Finally, we crossed LSL-iRFP mice with two GEM tumour models to assess if iRFP can be used to quantify tumour growth with a PEARL near-infrared imager. We chose two cancer models (lung and pancreas) where the assessment of tumour development in live mice is challenging due to inaccessibility for caliper measurement or physical barriers such as the rib cage. In the GEM lung model, mice harboring LSL- KRas^G12D^; Rosa26-LSL-MYC; LSL-iRFP were induced via intranasal Adeno-Cre administration. This will lead to lung specific Cre expression and induce iRFP positive lung tumours due to activation of mutant Kras and Myc. Over time the signal in the lungs increased, consistent with tumour growth (Fig. 4a and b). Since data acquisition of iRFP fluorescence can be carried out in rapid succession with exposure times as low as 500 ms, we were also able to obtain near real-time data (Supplementary video 1), which is not possible with imaging techniques like luciferase or PET scanning. These techniques require a longer acquisition time per image (typically 10 seconds to one minute for luciferase^14^) and - in the case of luciferase - imaging is further complicated by the substrate injection kinetics of luciferin^15^. At endpoint, lungs were resected and tumour presence was confirmed by histology (Supplementary Fig. 5).

For our second model, we turned to Tg^(Pdx1-cre)6Tuv/J^ (Pdx1-Cre) – a widely used allele for the induction of genetic alterations in the pancreas. Crossing onto an LSL-iRFP strain showed that iRFP is sensitive enough to image Pdx1-Cre activity in wild type mice, allowing us to visualize the pancreas in live animals (Supplementary Fig. 6, left). Next, we used iRFP to investigate where Pdx-1 Cre is active. As reported by Jax laboratories^16^, Cre-dependent recombination could be detected in the pancreas, the stomach and the duodenum. However, in addition to these sites, we also identified sporadic Cre activity in the liver (Fig. 4c). These data highlight the utility of iRFP as a sensitive tool to identify the location of Cre activity, and also suggest that Pdx-1 Cre activity in the liver should be taken into account – especially in studies measuring incidence of liver metastasis from Pdx1-Cre driven pancreatic primary cancers. Importantly, the signal in these otherwise normal mice did not change over time (Fig. 4d, wt1 and wt2).

Moving into a pancreatic tumour model, we introduced Cre-inducible versions of 172 H mutant p53 and oncogenic K-ras (the ‘KPC’ model^17^). We observed rapid tumour growth, as reported previously^17^, and an iRFP signal that increased over time to an endpoint significantly higher than the wild-type controls (Fig. 4d and Supplementary Fig. 6). Since the iRFP signal could already be measured in LSL-iRFP Pdx1-Cre animals and increased signal was observed prior to a palpable tumour from KPC mice with iRFP, we were able to use iRFP fluorescence to monitor and quantify tumour development more closely than with palpation alone, and were able to obtain data documenting tumour growth (Fig. 4d). Interestingly we also were able to detect papillomas in most (3 out of 4, Supplementary Fig. 6, red arrows) KPC mice. *In vivo* imaging also allowed us to identify a lung metastasis, which would not have been detected by palpation. The presence of these tumours was confirmed by imaging the individual organs post mortem (Fig. 4e). Guided by fluorescence imaging we were able to take both primary tumour and metastatic tissue into culture. As we have reported previously using overexpressed iRFP^3^, LSL-iRFP is an accurate marker of cell growth in culture, allowing us to detect an accelerated growth rate in the metastatic line compared to the primary tumour *in vitro* (Fig. 4f). To confirm that iRFP is a suitable marker for frozen sections, we scanned unfixed cryo sections on a LI-COR Odyssey scanner and verified that fluorescence is maintained in snap-frozen tumour sections (Supplementary Fig. 7). We were able to confirm that iRFP expression did not alter tumour histology. There was no difference in the histology of the lung tumours with and without iRFP (Supplementary Figs. 5 and 8) and both KPC with and without iRFP were histologically similar – with the detection of both moderately well differentiated tumours with some glandular features (Supplementary Fig. 9, upper panels) and tumours with a relatively more undifferentiated morphology (Supplementary Fig. 9, lower panels).

## Discussion

In this study we have shown LSL-iRFP to be a sensitive marker to identify recombination events. Conditionally activated iRFP can be quickly and reproducibly detected with a sensitivity that allows for an accurate assessment of the level of Cre activity in different organs. In the case of R26CreERT2 this may allow the use of a lowered dosage of tamoxifen, reducing potential adverse effects of the drug that otherwise may complicate the analysis of a phenotype.

We also show that a single allele of iRFP integrated into an endogenous locus is sufficiently bright to be detected in single cells by FACS analysis and that iRFP fluorescence is sensitive enough to monitor tumour progression in GEM models *in vivo*, in a rapid and cost effective manner. Importantly, iRFP does not alter the histology of the tumours in the models investigated here. Our data also demonstrate that iRFP fluorescence can be used to follow metastatic events in tissues distant from the primary site. In addition, this system allows direct transfer from *in vivo* experiment to primary tissue culture without the need to further manipulate the sample beforehand – and the ease and sensitivity of iRFP as a marker can allow for the detection of subtle but important differences in growth rates of cells derived from primary tumours or metastases. This technique does not add any additional steps to the standard culturing protocols and can be miniaturized, allowing the growth of organoids to be measured in real time and at high throughput. This is of particular benefit when working with primary cultures, where material is often limiting, expansion of cultures expensive and single cell counting very cumbersome.

We believe the superior optical properties of iRFP will be a valuable asset to overcome some of the complications inherent to imaging live animals and a powerful tool for measurement of the growth in complex 3D cell culture systems *in vitro*.

## Materials and Methods

### Genetically engineered mice, mouse procedures and organoid cultures

#### Generation of transgenic flies and fly husbandry

The full-length cDNA fragment encoding iRFP (derived from Addgene construct #31857) was cloned into the pUAST vector and injected into w−/− embryos using standard P element transformation techniques.

Flies were maintained on standard molasses medium in 12 hr light-dark cycles. UAS-iRFP or *w* ^*1118*^ (Bloomington #6326) as control flies, were crossed with Gal4 bearing virgin females at 25 °C. Progeny carrying either transgenes, or the single driver as control, were selected for imaging. GAL4 drivers used in this study were: CG-G4 (Bloomington #7011), elav-G4 (Bloomington #8760), tubulin-G4 (Bloomington #62712) and Armadillo-G4 (kind gift from Allison Gontijo).

For toxicity determination, 50 newly hatched larvae for each genotype were handpicked from agar-juice plates supplemented with yeast paste in quadruplicate and transferred to normal food. Adults were manually scored irrespective of sex.

Procedures involving mice were performed in accordance Home Office license numbers 60/4181, 70/8645 (Karen Blyth) and 60/4293 (Douglas Strathdee), carried out in-line with the Animals (Scientific Procedures) Act 1986 and the EU Directive 2010, and sanctioned by Local Ethical Review Process (University of Glasgow). Mice were housed on a 12/12 light/dark cycle and fed and watered ad libitum. To reduce the risk of autoflourescence, mice were moved to alfalfa-free chow (AIN-93M, DBM Scotland) for at least 5 days prior to imaging. No exogenous biliverdin was introduced in any of these experiments. Tamoxifen was injected intraperitoneally once (3 mg tamoxifen; equals low dose in manuscript) or 4 times (3 mg of tamoxifen for 1 day followed by 2 mg of tamoxifen for 3 days; equals high dose in manuscript). Hprt^tm1(CAG-LSL-iRFP)^ (LSL-iRFP) mice were developed in house as described in the paper. Gt(ROSA)26Sor^tm2(cre/ERT2)Brn^ (R26CreERT2)^13^ and the KPC model^17^ were previously described. All animals were genotyped by Transnetyx (Cordova, TN). Recombinant adenovirus expressing Cre was purchased from the University of Iowa gene therapy core facility. For Adeno-Cre installation Adeno-Cre was administered intranasally using the calcium phosphate precipitation method, as described previously^18^. Intestinal crypt cultures^19^ and pancreatic organoid^20^ cultures were performed as described previously.

### FACS analysis

FACS data were acquired on a BD LSR Fortessa flow cytometer running BD FACSdiva software (both from BD biosciences). Samples were excited with the UV laser (355 nm) for DAPI and the red laser (640 nm) for iRFP, with emission detected using 450/50 and 730/45 bandpass filters for detection of DAPI and iRFP, respectively. Live cells were identified by DAPI exclusion (DAPI added just prior to analysis, 1ug/mL final concentration) and median fluorescent intensity of iRFP in the live cell population in each sample was assessed. Data were analyzed using FlowJo X 10.0.7r2 (FlowJo, LLC). Livers were passed through a 70 µm cell strainer and FACS analysed. Primary pancreas samples were digested with trypsin to separate cells and subsequently treated as the liver cells.

Pancreatic organoids were resuspended in ice-cold PBS and mechanically separated, spun down and subsequently digested with dispase to remove matrigel, washed and passed through a 70 µm cell strainer.

### Imaging and quantification

Spectra in Fig. 1a were measured using a BioSpectrometer basic (Eppendorf). Images were taken by de-convolution microscope (Supplementary Fig. 1 left panel) Odyssey (LI-COR) (Figs 1b–f, 2b–g, 3a,d,g and 4c; Supplementary Figs 3, 4 and 7) or PEARL (LI-COR) (Figs 2a, 4a–f and Supplementary Fig. 5). Imaging parameters within series have been kept constant.

The fluorescence images in Supplementary Fig. 1 right were obtained on an Olympus IX81 inverted microscope controlled via the Volocity (Perkin Elmer) software package. A 660 nm emitting LED (Thorlabs M660L3-C1) was used to excite the iRFP emission through a custom filter cube consisting of a 655/40 excitation filter, a 685-Di01 dichroic filter and a 716/40 emission filter (all Semrock). The exposure time was adjusted to avoid saturation on the CDD camera and were typically 50 ms.

All Odyssey scans were prepared and quantified with Image Studio v5 (LI-COR) and displayed with false color LUTs. No exogenous biliverdin was introduced in any of these experiments.

Li-COR supplied filter sets and light sources were used for the Odyssey imaging systems. For excitation of iRFP, emission wavelength of 685 nm (700 channel) was used. The detection maxima is 730 nm and have a peak power rating of 0.5 watt.

### *In vivo* Imaging

All quantifications were performed with Image Studio v5 (LI-COR). Prior to imaging *in vivo*, mice were treated with depilatory cream (nair) to reduce scattering and absorption of light, as well as to reduce influence of coat color on individual images. Li-COR supplied filter sets and light sources were used for the PEARL imaging systems. The following laser was used for excitation of iRFP: The emission wavelengths are 685 nm (700 channel). The detection maxima is 730 nm and have a peak power rating of 0.5 watt. No exogenous biliverdin was introduced in any of these experiments.

### Data plotting

All data were plotted using Prism (Graph Pad) and Weibull growth models were used in Figs 2g and 4b. Figures were prepared using Illustrator (Adobe).

### Data Availability

The datasets generated during and/or analysed during the current study are available from the corresponding author on reasonable request.

## Electronic supplementary material

Supplementary Information
