# Supplementary material for: Development of an inducible mouse model of iRFP713 to track recombinase activity and tumour development in vivo
Source: Sci Rep. 2017 May 12;7:1837. doi: 10.1038/s41598-017-01741-0 (PMC5431786; doi:10.1038/s41598-017-01741-0)
Supplement: Supplementary file 1 — Supplementary Information [file 41598_2017_1741_MOESM1_ESM.pdf]

Development of an inducible mouse model of iRFP713 to track recombinase activity and tumour development *in vivo*.

Andreas K. Hock[1], Eric Cheung[1], Timothy J. Humpton[1], Tiziana Monteverde[1], Viola Paulus-Hock[2], Pearl Lee[1], Ewan McGhee[1], Alessandro Scopelliti[1], Daniel J. Murphy[1], Douglas Strathdee[1], Karen Blyth[1] and Karen H. Vousden[1,3]

[1]*Cancer Research UK Beatson Institute, Switchback Road, Glasgow, G61 1BD, UK*
[2]*Institute of Cancer Sciences, University of Glasgow, Switchback Road, Glasgow, G61 1QH, UK*
[3]*Corresponding author*

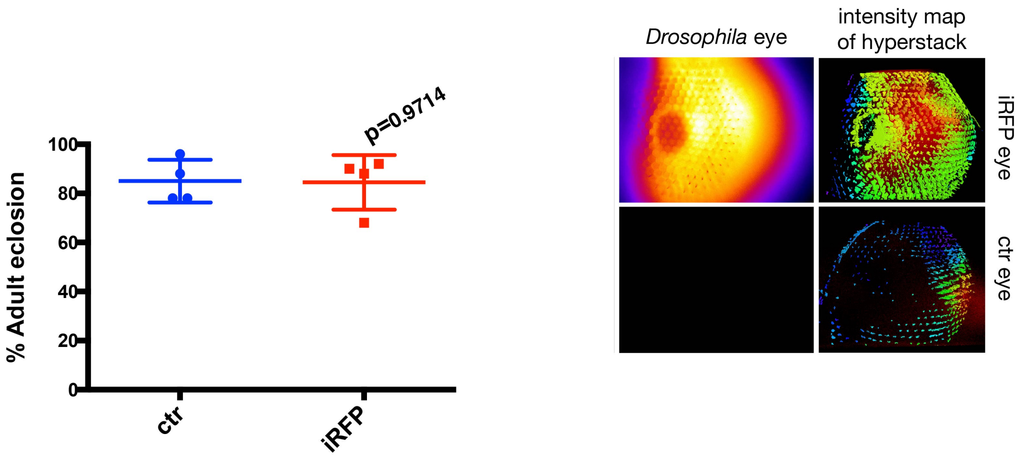

Supplementary Figure 1:
Left: Adult eclosion of control and iRFP Drosophola. Bars represents mean and SD.
Right: Microscopic analysis of Drosophila eye.

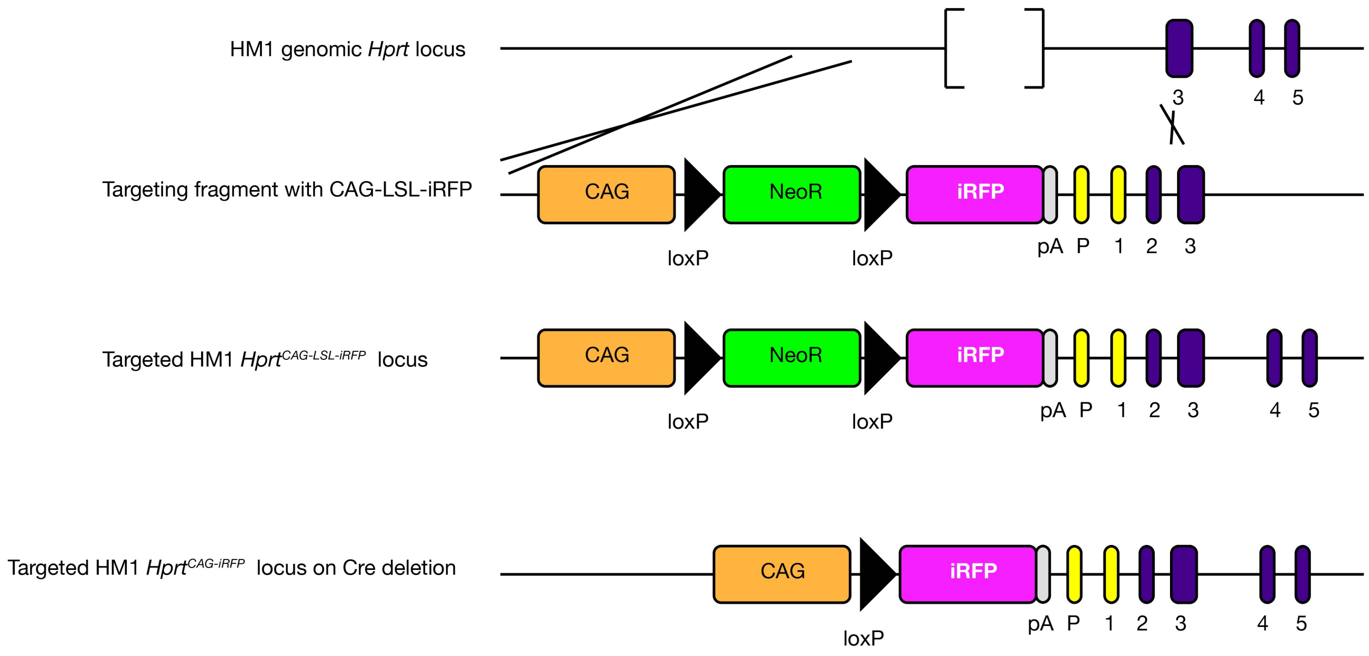

Supplementary Figure 2: Knock-in strategy of LSL-iRFP

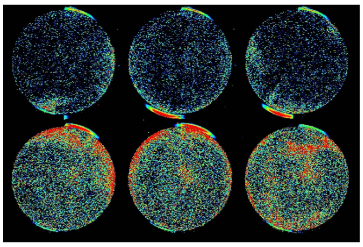

Supplementary Figure 3: LI-COR Odyssey scan of Cre induced mouse embryonic stem cells (mESCs). Deletion of the STOP cassette by transient expression of Cre in transfected mESC allows expression of iRFP and selection of clones.

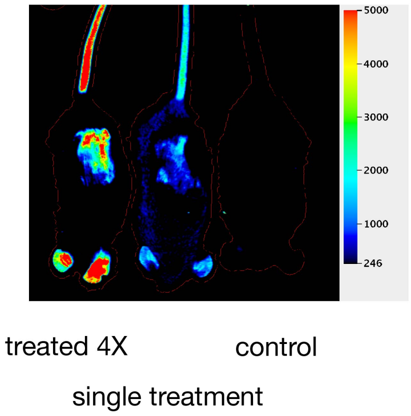

Supplementary Figure 4: Scan of tamoxifen induced mice.
Animals were induced as indicated and imaged on a LI-COR Odyssey.

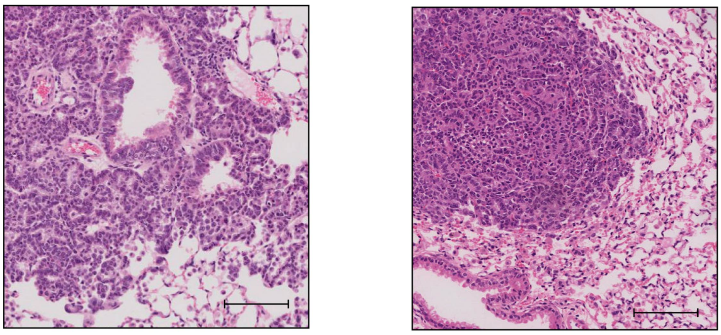

Supplementary Figure 5: HE stains of resected lung. Scale bar equals 100µm.

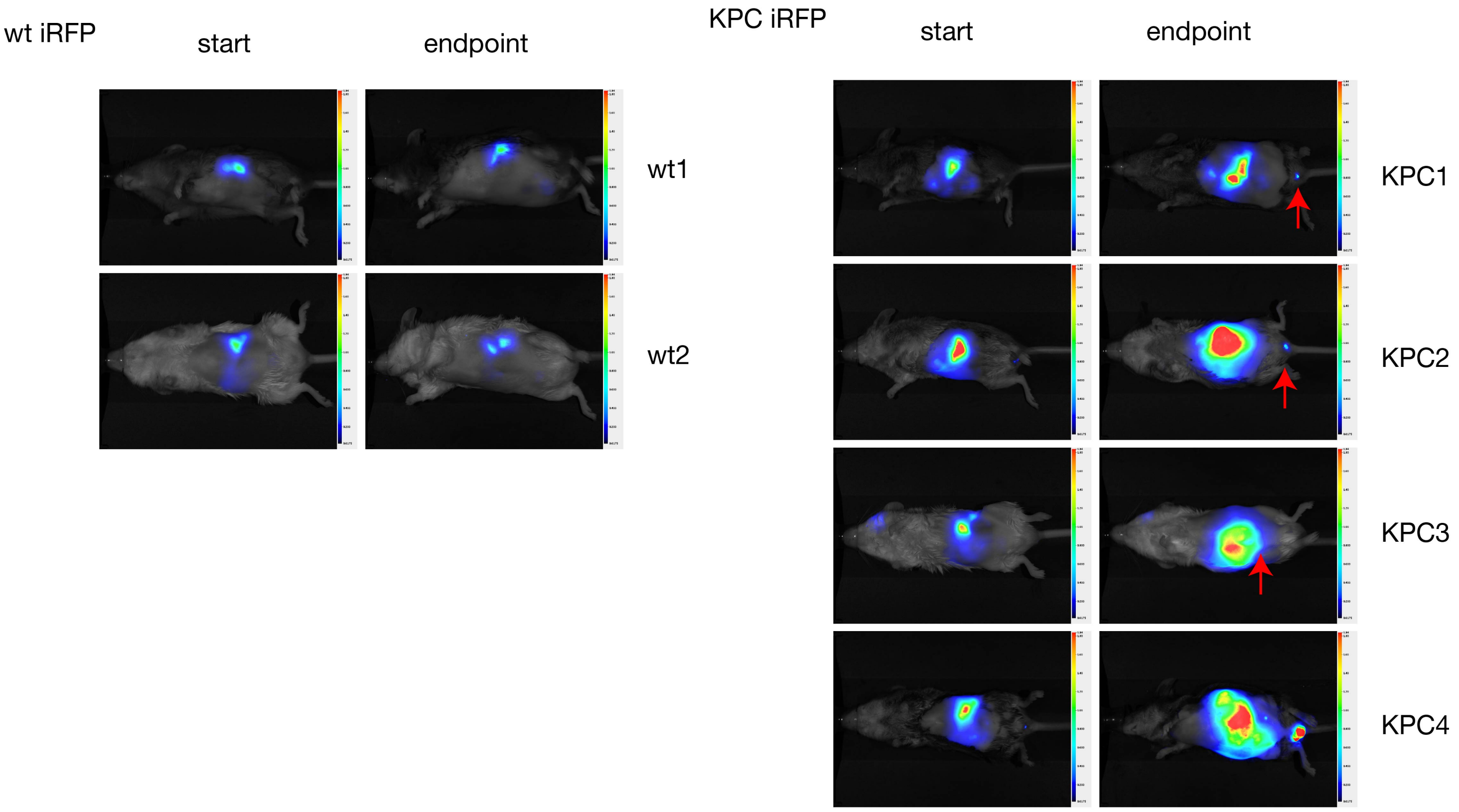

Supplementary Figure 6: LI-COR PEARL images of mice quantified in Fig. 4d. The red arrow highlights anal papilloma. Identical LUTs were used.

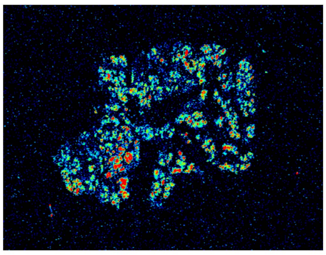

Supplementary Figure 7: LI-COR Odyssey scan of pancreatic tumor frozen section.

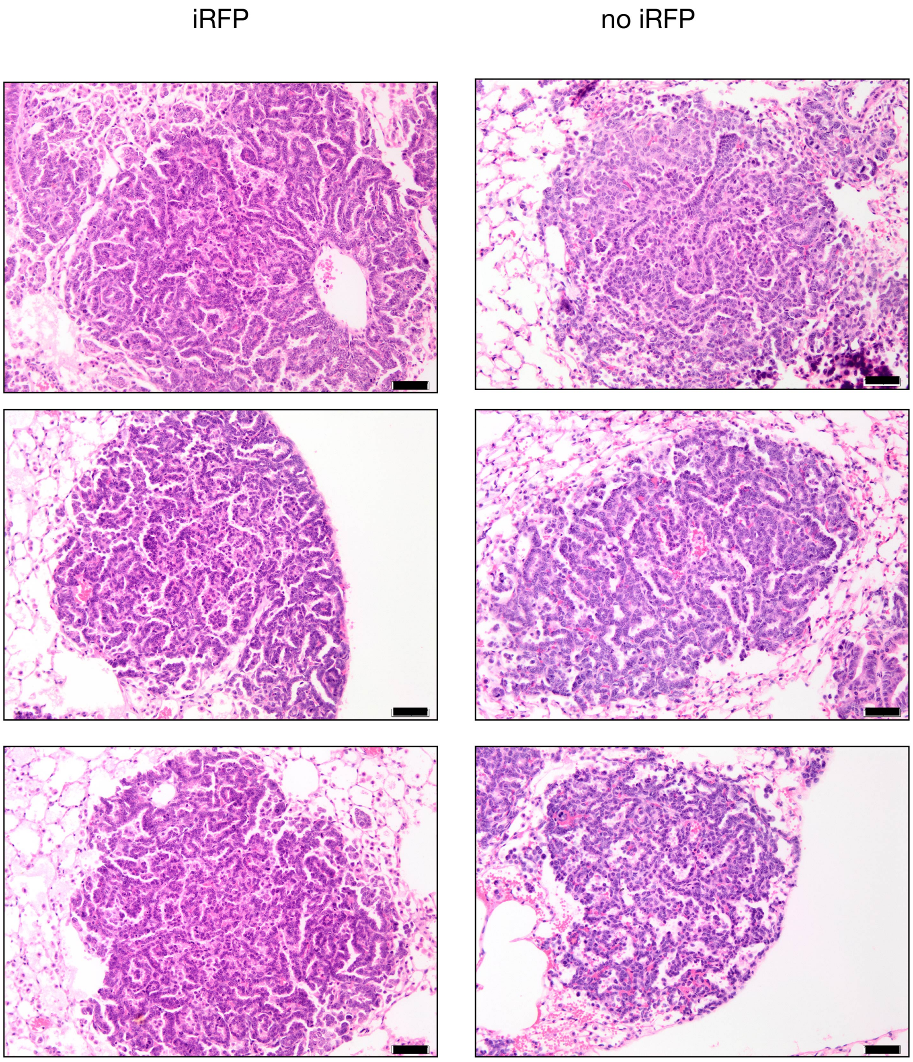

Supplementary Figure 8: Histological comparrrison of lung tumours with and without iRFP Different lung tumours from an animal with (left) and without iRFP (right) have similar histology. Bar is 500μm

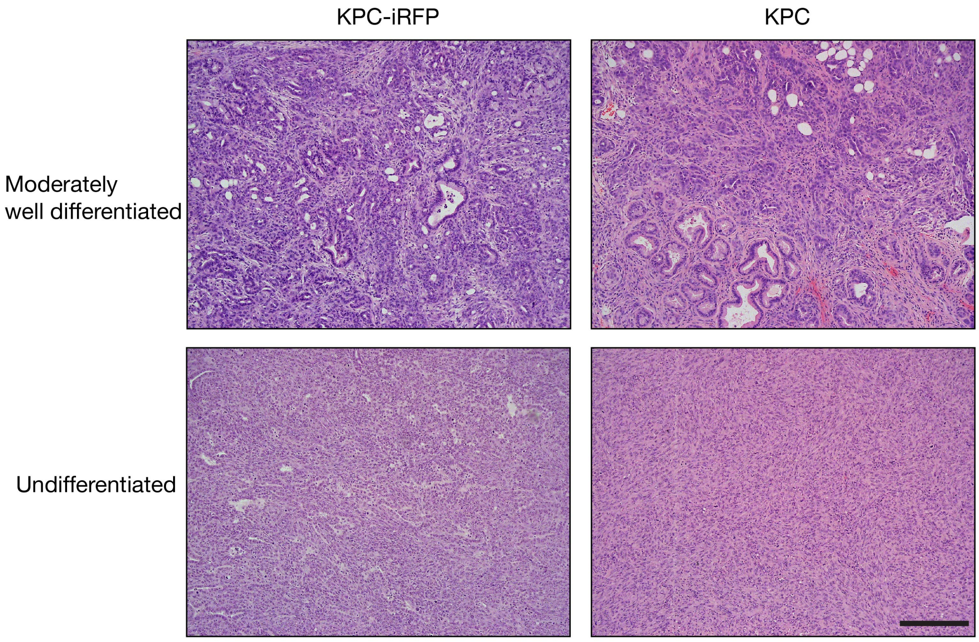

Supplementary Figure 9: Histological comparrrison of PDAC with and without iRFP
Both KPC with and without iRFP have similar histology of PDAC, such as moderately well differentiated with some glandular features (upper panels) and relatively more undifferentiated morphology (lower panels).
Bar is 20μm
